# Adolescent Idiopathic Scoliosis and Mental Health Disorders: A Narrative Review of the Literature †

**DOI:** 10.3390/children9050597

**Published:** 2022-04-22

**Authors:** Ioanna Mitsiaki, Athanasios Thirios, Eleni Panagouli, Flora Bacopoulou, Dimitris Pasparakis, Theodora Psaltopoulou, Theodoros N. Sergentanis, Artemis Tsitsika

**Affiliations:** 1 MSc Program “Strategies of Developmental and Adolescent Health”, School of Medicine, National and Kapodistrian University of Athens, 115 27 Athens, Greece; ioannamits1995@gmail.com (I.M.); dunapower@gmail.com (A.T.); elenpana@med.uoa.gr (E.P.); tsergentanis@yahoo.gr (T.N.S.); 2 Center for Adolescent Medicine and UNESCO Chair Adolescent Health Care, First Department of Pediatrics, “Agia Sophia” Children’s Hospital, School of Medicine, National and Kapodistrian University of Athens, 115 27 Athens, Greece; fbacopoulou@med.uoa.gr; 3 Pediatric Orthopaedic Department, Athens Medical Center, 151 25 Athens, Greece; dim_pasp@yahoo.gr; 4 Department of Clinical Therapeutics, “Alexandra” Hospital, School of Medicine, National and Kapodistrian University of Athens, 115 28 Athens, Greece; tpsaltop@med.uoa.gr

**Keywords:** idiopathic scoliosis, adolescents, mental disorders, eating disorders

## Abstract

Adolescent idiopathic scoliosis (AIS) is the most common type of scoliosis. The condition begins in puberty, affects 1–4% of adolescents, and disproportionately affects young women. Our aim was to comprehensively examine the association between AIS and risk for depression, anxiety, eating disorders, psychotic disorders, and personality dysfunctional mechanisms. Methods: Literature review of related articles published in PubMed, Google Scholar, and Scopus up to 15 July 2021. Results: A total of 30 studies were deemed eligible, examining the effects of AIS upon mental health, and using appropriate psychometric inventories. Studies highlighted the association of brace treatment with elevated anxiety. In addition, mental health conditions and traits (e.g., anxiety and depressive symptoms, neuroticism) were detected more frequently amongst AIS patients compared to healthy controls. Conclusions: AIS represents a risk factor for mental health disorders. More longitudinal studies, utilizing accurate psychometric instruments, are warranted, to reveal the current impact of AIS on the mental health of youngsters, along with the tailoring of well-targeted interventions to reduce the burden of mental health issues in adulthood.

## 1. Introduction

Scoliosis is defined as a three-dimensional (3D) structural deformity of the spine and for practical reasons it is widely diagnosed radiographically, on the basis of the measurement of the Cobb angle. A Cobb angle is used as a measurement of the degree of spinal curvature; thus, scoliosis is present in case of at least 10 degrees of deviation [1]. In most cases, the etiology of scoliosis is multifactorial, and since 1922 these patients have received the diagnosis of idiopathic scoliosis [2]. Idiopathic scoliosis (IS) is classified into three categories, depending on the peak period of onset: the infantile, which occurs under the age of three; the juvenile, from five to eight years old; and the adolescent, which affects teenagers from 10 to 19 years old [3]. The latter accounts for 80% of idiopathic scoliosis cases, and a meta-analysis on its prevalence has shown a range from 0.47 to 5.2%, after school-screening [4].

Adolescent idiopathic scoliosis (AIS) involves high possibilities of progression, primarily amongst girls during the growth spurt at puberty [5]. From mild to moderate deformities, bracing is the most common type of conservative treatment aiming at controlling such progression, whereas curves greater than 40 degrees require surgery [6]. When untreated, idiopathic scoliosis may lead to severe trunk deformities, which might cause limitations to the general quality of life and well-being of adolescents with idiopathic scoliosis [7]. However, regardless of the treatment prescribed, there is a critical threshold in the current literature between 30° and 50° associated with a high risk of long-term curve progression [8]. Therefore, in many cases, adolescent idiopathic scoliosis progresses in adult life and is usually combined with significant clinical problems [9].

An accurate diagnosis of AIS includes a combination of individual factors concerning the Cobb angle, the angle trunk rotation (ATR), the sagittal balance, the Risser sign, which indicates the level of skeletal maturity, and the pulmonary function measured by spirometry [10,11]. Hence, the goals of conservative treatment, from observation to bracing and physiotherapeutic scoliosis-specific exercises (PSSE), have been accordingly adapted, in order to control curve progression, to avoid surgery, and to prevent or treat respiratory dysfunction [12].

Bearing in mind the critical period of the appearance of AIS, many studies have marked the psychological impact of various parameters regarding AIS [13]. Bracing, poor body image, due to the deformities, and pre- and post-operative pain of surgery have been outlined as stressful factors for youngsters, in addition to the need for psychological adjustment and compliance to the treatment [14,15].

In parallel, childhood and adolescence is the core risk period for the development of mental health issues, ranging from transient mild symptoms to more severe disorders. Anxiety disorders have been reported as the most common condition (31.9%), followed by behavior disorders (19.1%), and mood disorders (14.3%) [16]. Anxiety has been perceived as a comorbid condition with many mental disorders, such as psychosis [17], but also with medical conditions such as musculoskeletal conditions [18]. Additionally, eating disorders represent an important issue amongst teenagers, with high incidence rates [19]. Furthermore, personality disorders and personality traits such as neuroticism, dependency, and introversion have been investigated as predictors for maladaptation and future psychopathology [20,21].

Consequently, this review of the literature aims to evaluate the association between AIS and mental health disorders, on the basis of specialized instruments. 

## 2. Materials and Methods

### 2.1. Literature Search Strategy

A literature search was performed on 30 May 2021 in three different databases (PubMed, Scopus, and Google Scholar). For the search, various key-words were used: idiopathic scoliosis, teenagers, youngster, adolescence, young adults, mental disorders, psychopathology, eating disorders, anorexia, bulimia, mood disorders, depression, emotional reactions, cyclothymic disorder, premenstrual dysphoric disorder, anxiety, and stress. Those terms were used in pairs, using “AND” or “OR”, in order to receive specified results. 

### 2.2. Inclusion Criteria

This review aims to investigate the association between AIS and mental health disorders, or indices of psychopathology, in adolescence or in adulthood. For this reason, eligible articles had to meet the following criteria:

1. Studies had to report on adolescents or young adults with a diagnosis of AIS in their puberty, in terms of retrospective studies.
2. Studies could be purely based on AIS, present results on a subgroup analyses on AIS, or encompass AIS cases as the majority of the study sample.
3. Studies had to provide data about the correlation between AIS and mental health disorders, as defined by appropriate psychometric instruments. Any strategy to diagnose AIS was deemed eligible.
4. Prospective cohort/cross-sectional/case-control/retrospective/large-data based epidemiological studies/reliability and validity analyses of questionnaires were included.
5. The article was written in English language.
6. There was no restriction in publication year.

### 2.3. Exclusion Criteria

Articles meeting the following criteria were excluded from the review:

1. Articles evaluating different aspects of patients’ well-being and psychological function utilizing generic inventories.
2. Articles exclusively on other types of scoliosis were also excluded (congenital, neuromuscular, degenerative, Scheuermann’s kyphosis, or syndromic)
3. Literature reviews; however, these were screened for relevant resources not found via search terms.

### 2.4. Data Abstraction

Numerous studies were retrieved from the databases. After the first check, a great number was excluded by title or abstract. The studies that were considered eligible were evaluated in full-text and separately tabulated on the basis of the mental health condition they assessed. From each study, analytic characteristics were extracted concerning the study design, the sample size, the percentage of males, study population, assessment of scoliosis, evaluation of mental health disorder, treatment prescribed, severity of the main curve, and the main findings.

## 3. Results

### 3.1. Study Characteristics

A total of 30 studies, from 1992 to 2021, were included in the present review [22,23,24,25,26,27,28,29,30,31,32,33,34,35,36,37,38,39,40,41,42,43,44,45,46,47,48,49,50,51]. Most of those included were cross-sectional, (15 of 30), including eight from Poland, two from China, two from United Kingdom, two Italian, and one from Spain. The others were mainly prospective cohort studies, including seven ranging from Poland, USA, and Taiwan to UK and Sweden. Among the remaining ones, there were four case-controlled studies, one registered audit from the UK, an Iranian validation study, and one clinical trial from Spain. The percentages of male patients were significantly lower (range: 0–41%) and only one study was exclusively based on male subjects with scoliosis [50]. Most cases of idiopathic scoliosis detected were adolescent type, with an overall moderate severity (20–40°) treated by brace. With regards to mental health disorders, nine studies examined stress/anxiety, three studied depressions, eight studied jointly the aforementioned conditions, four examined eating disorders, five studied personality pathology, and two studied schizophrenia. The study of Oh et al. [43] was added in both the latter categories. Demographic characteristics are represented in Table 1.

The tables present the studies on the association between AIS and stress/anxiety, depression, joint examination of depression and anxiety, eating disorders, personality disorders and traits, and psychotic disorders (Table 1 and Table 2).

### 3.2. Results of Individual Studies

The majority of the studies found during the literature research marked the association between AIS and increased levels of stress/anxiety. Nine studies from 2001 to 2018, indicated an overall moderate to low stress amongst a large part of the adolescent population treated by brace, surgery, or physiotherapy (Table 1 and Table 2). On the one hand, Misterska [25,26], as well as Motlagh [27], Kotwicki [28], and Leszczewska et al. [29], using the same inventory of BSBQ, concluded that the brace increased the level of stress in comparison to the stress induced by the deformity alone. Similarly, Glowacki et al. [22], evaluated the anxiety of braced adolescents by STAI-C inventory, showing a constant brace-related anxiety during a 12-month observation period. Two additional parameters that Mistreska et al. pointed out pertained to the statistically significant correlation between apical translation and stress level, as well as the age of initiation of the conservative treatment [24]. However, from the studies jointly examining depression and anxiety (Table 1 and Table 2), Sanders et al. found that 32% of patients with AIS exhibited significant psychological distress, regardless of the type of treatment. In this study, anxiety represented the most common concern for teenagers, with a similar occurrence as in pediatric cancer and heart transplant patients [39]. Supportive evidence was provided by a recent large-database epidemiologic study from 2012 to 2016, showing that among the overall prevalence of 7% of mental health disorders in AIS patients, the highest percentages were attributed to anxiety [41].

On the other hand, adolescents with severe scoliosis requiring surgery, experienced a significant increase in anxiety levels postoperatively, which was explained by the pain intensity [21]. Rullander et al. marked a similar correlation between the level of pain and anxiety (*p* = 0.03) in Trauma Symptom Checklist for Children, reporting that the studied adolescents had higher stress symptoms before, than after, surgery [40]. The higher symptoms of anxiety in AIS surgery patients compared to healthy controls was also demonstrated by the study of Duramaz et al.; although, indicating a notable decrease in such symptoms after the spinal correction [35]. The stressful experience of scoliosis surgery was also indicated by the scores of HADS anxiety inventory in another study, concluding that operative treatment resulted in elevated levels of anxiety [38]. From a similar biopsychosocial perspective, Wong et al. demonstrated a remarkable prevalence of back pain amongst AIS patients, regardless of a surgical treatment. The high prevalence of chronic back pain increased the odds for anxiety, depression, insomnia, and daytime sleepiness [36]. The morbidities for anxiety and depression were also investigated by Wang et al., in patients with AIS and their parents, showing that only 7.3% had scored within the cutoff point of 10 that denoted probable major depressive disorder and 3.2% for severe anxiety. However, this cross-sectional study showed that the morbidities of parental depression and anxiety were 14.1%, significantly higher than those in the control group, proposing a causal relationship between parents’ mental disorders and adolescents’ general distress [34].

Table 2 summarizes the characteristics of the studies focusing on the association between AIS and depression. The depressive symptoms, when evaluated by Lin et al., showed an overall medium extent, which was significantly higher amongst female patients. The greater presence of such symptoms in AIS than in juvenile IS patients reflected the vulnerability of adolescence to mental health issues. The parameters positively associated with the severity of depression were the duration of brace treatment and the severity of the main curvature, as was already reported above within the studies examining the anxiety levels in braced patients [31]. Indeed, depression was proved to be more prevalent amongst scoliotic patients during a 5-year follow up; specifically, Chang et al. found higher hazard ratios of depressive disorders in patients with scoliosis and mostly amongst young adults and the middle-age [32]. In parallel, the risk of depression was also demonstrated by Baird et al., who found that 18% of AIS patients had scores worthy of further assessment for a potential diagnosis of depression [37]. Finally, the only study in the current literature that evaluated depression in young women with AIS using the Beck Depression Inventory marked a greater tendency for depression in female patients with mild and moderate deformities [33].

Regarding the matter of eating disorders, variable results have been published (Table 2). Alborghetti et al., using the Structured Clinical Interview for DSM-1 Disorders (SCID-1), showed a significantly higher prevalence of eating disorders in the scoliosis group when compared to an Italian female population (9.2% for anorexia nervosa, 7.7% for bulimia nervosa, and 5.3% for EDs). Interestingly, it was also highlighted that only girls with AN had a significantly higher degree of deviation of the vertebral column than adolescents without AN, F (1, 76) = 3.87, *p* < 0.05, indicating an association between the severity of scoliosis and the presence of AN [42]. Similarly, Smith et al. demonstrated the relationship between a diagnosis of AIS and anorexia nervosa, indicating that more than a half of the studied patients had BMI scores within the range considered to be anorexic [43]. On the other hand, Zaina et al., using the Eat-26 questionnaire, showed a low prevalence of eating disorders in AIS female patients. The significantly lower BMI remained present, but it was considered typical of scoliosis patients [44]. From a common perspective, Smith et al. showed that, notwithstanding the typically lower body mass index (BMI) of the studied group, the prevalence of eating disorders remained very low and similar to the general population [45].

Regarding personality pathology, results from the included studies suggested a pattern of neurotic personality traits (Table 2). These findings were demonstrated by the studies of Oh and Misterska et al. Both authors, using personality inventories, indicated higher scores in the neurotic scales amongst AIS patients compared to healthy controls [47,50]. Oh et al. [50], assessing males during the end of puberty obtained more accurate indices of personality disorders, using a conventional type of the Minnesota multiphasic personality inventory test, which is one of the most widely used psychological assessment tools [50]. Similar scores were demonstrated by Misterska et al., reporting higher scores in the neurotic scale for the group treated surgically, whereas the AIS group under brace treatment had higher scores in the manic scale [47]. Matsunaga, on the basis of a clinical trial, confirmed the trend towards neuroticism, investigating the impact of brace therapy in the personality functioning and emotional responses of the studied adolescents. Although these traits seemed to be modifiable, it is important to notice that just one month after the start of therapy, the MPI assessment showed that 25% of the studied population encountering neurotic characteristics, compared to 2% before bracing [48]. Additionally, Matsunaga [48], along with the studies of Leak [46] and D’Agata et al. [49], have jointly unveiled a passive-introverted tendency among AIS patients, connected in all cases with brace-therapy and the challenging task of compliance.

Finally, two studies evaluated an association between psychosis and scoliosis (Table 2). The study of Oh et al. is twice referred to, but alternatively interpreted, since MMPI is identified, as well as symptoms associated with psychosis. Significantly higher scores in the psychopathy set including schizophrenia were reported among the 213 examinees (*p* = 0.010) [50]. Likewise, an increased risk of schizophrenia in patients with idiopathic scoliosis was detected by Malmqvist et al., over a median follow-up time of 9.5 years after the AIS diagnosis. The odds ratio for schizophrenia was significantly higher in patients with IS (HR, 1.52; 95% CI, 1.03–2.23), suggesting a possible link between these two conditions [51].

## 4. Discussion

The present literature review is an attempt to summarize all the available studies designed to investigate the possible relationship between AIS and mental health disorders. The existing studies are mainly cross-sectional, (15 of 30) and based on questionnaire assessments, with small consecutive samples. In terms of stress/anxiety evaluation, BSSQ was the most widely used tool; only one study in the current bibliography used a GAD inventory [34], and two used the state-trait anxiety inventory [23,35]. The overall stress in AIS population was low to moderate; a large-database epidemiological design showed a positive association between AIS and mental health conditions, especially anxiety [41]. Accordingly, an assessment of youthful scoliotics with BASC-2 inventory indicated similar results of clinically significant distress, regardless of the type of treatment prescribed [39]. An additional finding related to surgically-treated patients that needs further investigation pertains to the positive correlation between the pains these patients experience and preoperative anger, social problems, and attention problems [40].

The current studies and reviews underscore the need to attend to the psychological assessment of AIS patients, and provide much supportive evidence of the stressful and demanding experience of AIS. Physical complaints, lower self-esteem, higher depression scores, feelings of shame and body inferiority, and suicidal thoughts have been detected and raise concern [52,53]. Accordingly, in our review the association between AIS and depression was highlighted [32,33].

Body dissatisfaction, which has been bibliographically referred to as a major psychological effect of AIS [54], is also connected to eating disorders [55]. In the present review, an Italian study demonstrated a higher prevalence of EDs in scoliotic women [42]. The EAT-26 [44] and eating disorder examination questionnaire (EDE-Q) [45] evaluation, however, provided different results, showing a lower incidence of EDs in female scoliosis patients than in the general population. In any case, the scoliosis group weighed less and had lower BMI scores, and the possible eating pathology amongst this population has to be further investigated on the basis of larger sample sizes and clinical interviews.

As for personality pathology, previous findings have outlined that the threat to body image can lead some female patients to develop the defense mechanism of denial of their condition [55], with feelings of shame and difficulties in social integration [56]. In parallel, brace-treatment and the need for compliance may evoke neurotic behaviors, due to the insecurity and fear for the treatment result, and reverse the patterns of relating and interacting between family members [57]. Finally, a possible association between idiopathic scoliosis and schizophrenia was supported by two studies [50,51]. The high psychometric properties of the Minnesota Multiphasic Personality Inventory (MMPI) for detecting psychopathic traits [50] and the large-database retrospective design have to be considered as strengths of these two designs. However, limitations exist concerning the study population, where only males from the military conscription examinations were included [50]. In any case, there is much space for further investigation to detect personality and psychotic disorders in cases of AIS; conducting large cohort studies enrolling women and men.

Future studies should be distinctly directed to the mental health of adolescents and adults with scoliosis, in terms of large-data based epidemiological studies and systematic reviews. In clinical research, adolescents should be addressed with specific screening tools along with psychosocial interviews, such as HEEADSSS, which allows the clinicians to comprehensively evaluate mental health in puberty and indicate risk and protective factors [58]. Randomized clinical trials should also be conducted, in order to evaluate the effectiveness of psychoeducational and psychotherapeutic interventions on adolescents with psychological distress, due to their deformity. Consequently, it turns out that psychological support should constitute an integral part of the overall therapeutic protocol of AIS, in order to address the growing need for psychosocial support of scoliotic patients and to reduce the burden of mental health issues in the future.

This review has some limitations; studies on overall quality of life and psychological well-being were not included. Moreover, especially in case of depression and psychosis, we could have included studies with an admixture of AIS cases with other types of scoliosis. However, no such data were clearly defined and, due to the high prevalence of AIS, we could assume that the largest part of the studied population was diagnosed with AIS with a continuation in adulthood.

## 5. Conclusions

The findings of the present review suggest associations between AIS, depression, anxiety, and neuroticism. Future research should be promptly redirected to a screening perspective for mental health in AIS subjects, with the design of multidisciplinary interventions.

## Figures and Tables

**Table 1:** Demographic characteristics of the included studies.

Author (Year)	Region, Country	Study Period	Study Design	Sample Size	Number of Patient with AIS	Perc Ent Age of Males	Mean Age (SD)	Age Range	Study Population
Studies on the association between AIS and stress/anxiety									
Glowacki 2013 [22]	Poland	NR	Prospective cohort study: three clinical and questionnaire assessments	36	36	0	1st evaluation: 13.4 (1.7) 2nd evaluation: 14.0 (1.9) 3rd evaluation: 14.4 (1.7)	10–16	Female patients with AIS all recruited from one center
Lamontagne 2001 [23]	USA (82% Cau-casian, and 18% African-American.)	NR	Prospective cohort study	74	74	26%	NR	11 to 18 years	Convenience sample of adolescents who entered a southeastern hospital for elec-tive major orthopedic surgery for repair of idiopathic scoliosis and their parents
Misterska 2011 [24]	Poland	NR	Cross-sectional study	69	69	0	Patients treated operatively: 16.1 (1.5) Patients treated conservatively: 14.8 (1.4)	12 to 18 years	Female patients recruited consecutively, all treated in the Pediatric Orthopedics and Traumatology Clinic at the Poznan University of Medical Sciences by the same doctor
Mistreska 2011 [25]	Poland	NR	Cross-sectional study	64	64	0	Urban population: 13.9 (1.56) Rural population: 14.7 (1.49)	10 to 17 years	Female patients treated by the same orthopedic surgeon in the Pediatric Orthopedics and Traumatology Clinic
Misterska 2012 [26]	Poland	NR	Cross-sectional	63	63	0	14.12 (10.99)	10 to 17 years	Pairs of parents and female patients with AIS treated for a minimum of 1 month by the same orthopedic surgeon. Patients were recruited from 1 center
Motlagh 2018 [27]	Iran	NR	Validation study of the Persian versions of Bad Sobernheim Stress. Questionnaire-deformity/brace in adolescent idiopathic scoliosis	53	53	28.3%	13.47 (1.78)	10–16	Patients attending two spine canters in Tehran, Iran for a regular brace check-up
Kotwicki 2007 [28]	Poland	NR	Cross-sectional	111	111	0	14.2 (2.2)	10–20	Consecutive female patients
Leszczewska 2012 [29]	Poland	NR	Cross-sectional	73	73	12.32%	13.9 (2.1)	9–18	Children and adolescents undergoing conservative treatment
Zimoń 2018 [30]	Poland	From 3 September 2013 until 27 February 2014	Cross-sectional	63	63	14.28%	14.7 (2.2)	9–17	Patients treated in the Department of Pediatric Rehabilitation for at least 3 week
Studies on the association between AIS and depression									
Lin 2019 [31]	China	NR	Cross-sectional	208	112	AIS patients: 14.28% JIS patients: 26.04%	13.7 (2.2)	10–16	Cases of hospital patients with JIS and AIS from 2010 to 2016
Chang 2016 [32]	Taiwan	NR	Retrospective cohort study	8454	1409	31.9%	NR	18–40 (45.4%)	Study Cohort: patients with diagnosis of scoliosis from Longitudinal Health Insurance Database (LHID 2005) Comparison cohort: age and gender matched control subjects selected via random sampling
Płaszewski 2014 [33]	Poland	NR	Registry-based, cross-sectional study with retrospective data collection	144	68	36.7%	30.11 (4.11) Mean age at diagnosis: 10.5	24–39 Age range at diagnosis: 9–16	Participants from the population of subjects examined for scoliosis at the centre of Corrective and Compensatory Gymnastics, Bielsko-Biala, Poland
Studies jointly examining the association of depression and anxiety with IS									
Wang 2019 [34]	China	Between April 2017 and March 2018	Cross-sectional descriptive	149	64	7.8%	14.3 (2.2)	11–18	Patient–parent pairs
Duramaz 2018 [35]	Turkey	Between June 2014 and July 2015	Prospective case-control study	93	41	29.3%	15.3 (1.5)	12–18	Consecutive patient-healthy controls
Wong 2019 [36]	China	between December 2016 and July 2017	Prospective cross-sectional study	987	987	27.15%	14.7 (1.8)	10–18	Patients recruited from a tertiary scoliosis outpatient clinic in Hong Kong
Baird 2020 [37]	UK	between 6th May 2019 and 8th Oct 2019	Registered audit study	33	33	27.2%	14.5 (1.4)	12–17	Patients with an established diagnosis of AIS referred to a single spinal deformity centre in the UK
O Ryan 1999 [38]	UK (White 92.9% Asian 7.1%)	from September 1998 to March 1999	Exploratory, longitudinal prospective	28	28	17.9%	16.23 (2.26)	12.08–19.11	Patients having corrective surgery at Adolescent unit of an NHS national orthopaedic hospital.
Sanders 2018 [39]	USA	Between September 2014 and June 2015	Prospective cohort study	92	92	NR	Observation group: 14.2 Surgical group: 14.7 Brace group: 13	12–21	Adolescent patients
Rullander 2016 [40]	Sweden	NR	Prospective quantitative cohort study	37	37	13.5%	Girls: 15.8, boys: 16.1	13–18	Adolescents consecutively recruited from four Swedish spine centres
Lee 2021 [41]	Korea	From 2012 to 2016	Large-database epidemiologic study	1,047,603 ± 34,534 (mean number in each dataset)	7409 ± 158 (mean number for each year)	41% (for year 2016)	NR	10–19	Patient sample data from the Health Insurance Review and Assessment Service- age-matched controls: healthy children in the dataset
Studies on the association between IS and eating disorders									
Alborghetti 2009 [42]	Italy	NR	Cross-sectional	207	207	0	14.82 (1.40)	12–19	Female patients recruited from private and public orthopaedic rehabilitation institutes
Smith 2002 [43]	United Kingdom	NR	Cross-sectional	44	44	0	16	13–19	AIS female patients who attended the clinic
Zaina 2013 [44]	Italy	NR	Cross-sectional	187 AIS patients 93 controls	187	0	15.2 (2.5)	NR	Female patients under treatment and healthy schoolgirls without scoliosis as controls
Smith 2008 [45]	United Kingdom	NR	Cross-sectional	192	76	0	15.8	11–19	A scoliosis group recruited from outpatient clinics held at a regional treatment center for spinal deformity, a diabetes group and healthy school girls as controls
Studies on the association between IS and personality pathology									
Leak 1992 [46]	United States (White: 70.00% Hispanic:13.75% Black: 5.00% Indian: 3.75% Other: 7.50%)	from May to September 1990	Case-control	80	40	0	NR	13–16	Female patients recruited from the clinic and private practices of two orthopedic surgeons at Cornell Medical Center’s Hospital for Special Surgery in New York City. control group drawn from parochial schools in the same area
Misterska 2010 [47]	Poland	NR	Case-control study	104	69	0	NR	12–18	Female students treated in the Pediatric Orthopedics and Traumatology Clinic in the Poznań University of Medical Sciences by the same doctor
Matsunaga 2005 [48]	Japan	NR	A clinical trial	145	145	0	12.4	11–16	Female patients at our outpatient clinic
D’Agata 2017 [49]	Spain	NR	Cross-sectional	43	43	9.3%	14.3 (2.23)	10–19	Patients who visited the outpatient consulting clinic recruited consecutively for 1 year
Oh 2013 [50]	Korea	From April to November 2011.	Case-control study	213	105	100	19	NR	Males tested at the Seoul regional Military Manpower Administration and a normal volunteer group
Studies on the association between AIS and psychosis									
Malmqvist2019 [51]	Sweden	NR	Retrospective case-control study	373,902 (patients and controls)	3702	NR	14.3 (1.94) at diagnosis of AIS 10 at diagnosis of JIS	10–19	Swedish adolescents collected from the National Patient Register, diagnosed with IS between 1997–2015 and matched controls
Oh 2013 [50]	Korea	From April to November 2011.	Case-control study	213	105	100	19	NR	Males tested at the Seoul regional Military Manpower Administration and a normal volunteer group.

**Table 2:** Main Findings of the included studies.

Author (Year)	Sample Size	Number of Patient with AIS	Definition of Scoliosis	Curve Severity (Mean Cobb Angle, SD)	Treatment of Scoliosis	Definition of Anxiety	Main Findings of the Study
Studies on the association between AIS and stress/anxiety							
Glowacki 2013 [15]	36	36	Adolescent idiopathic scoliosis- radiologically and clinically assessed	27.1° (5.1) in the first and 24.9° (9.1) in the final examination.	brace	Trait version of the State-Trait Anxiety Inventory for Children (STAIC-trait)	1st-2nd-3rd evaluation: 83.4%, 91.7%, and 91.7% AIS females experienced low or medium anxiety, respectively. High anxiety was indicated in 16.6%, 8.3%, and 8.3% of study participants during the 1st, the 2nd, and 3rd evaluations. Anxiety levels were related during the 1st and the 2nd evaluation to the monthly duration of orthosis wearing (rs = 0.34 and rs = 0.33, respectively)
Lamontagne 2001 [16]	74	74	Adolescent idiopathic—established diagnosis	Severe scoliosis ≥ 40° (assumed by the type of treatment i.e., major orthopedic surgery)	surgery	Spielberger’s (1983) State-Trait Anxiety Inventory(STAI)	Children’s preoperative anxiety levels (M = 33.8, SD = 5.1) significantly increased on the second postoperative day (M = 38.8, SD = 7.2; F [1.70] = 21.9, *p* < 0.01). Children’s postoperative levels of anxiety were positively correlated with both day 2 pain (r = 0.55, *p* < 0.01) and day 4 pain (r = 0.37, *p* < 0.01).
Misterska 2011 [17]	69	69	Adolescent idiopathic- x-ray examination	Patients treated operatively: 54.6° (9.0) Patients treated conservatively: 27.7° (7.5)	35 patients treated by Chêneau brace the other 34 subjects were treated operatively and, after correction of scoliosis with thoracoplasty, wore a brace for 12 weeks during the postoperative period	Polish versions of the Bad Sobernheim Stress Questionnaire-Deformity and the Bad Sobernheim Stress Questionnaire-Brace	Brace increases stress moderately in both groups. The two groups have significant statistical differences, solely in relation to stress levels, due to body deformation (*p* = 0.004), where the group treated surgically reported higher stress levels. Age at initiation of treatment increases the stress levels of patients treated with a brace (*p* = 0.029). Significant statistical correlation (*p* = 0.008) pre-operatively between the degree of translation and stress levels due to body deformation in patients treated conservatively.
Mistreska 2011 [18]	64	64	Adolescent idiopathic scoliosis- Cobb angle evaluation	Urban population: 26.8° (5.6) Rural population: 28.2° (4.3)	Chêneau Brace	Polish version of the Bad Sobernheim Stress Questionnaire-Deformity and the Bad Sobernheim Stress Questionnaire-Brace	Brace wearing resulted in increased stress level when compared to the stress induced by the deformity alone in both subgroups: urban (*p* < 0.001) and rural (*p* = 0.002) environments.
Misterska 2012 [19]	63	63	Adolescent idiopathic scoliosis evaluated by radiographs taken on the same day as the questionnaires were filled in, in an anterior-posterior projection	28.52° (7.24)	Chêneau Brace	Bad Sobberheim Stress Questionnaire-Deformity and t Bad Sobberheim Stress Questionnaire-Brace	Patients experienced a moderate level of stress connected with conservative treatment and a low one related to perceived trunk deformation (*p* < 0.001). An adverse correlation between the apical translation and stress levels connected to body disfigurement due to wearing a brace (rs = −0.38, *p* = 0.002; rs = −0.30, *p* = 0.015, respectively)
Motlagh 2018 [20]	53	53	Adolescent idiopathic scoliosis measured by Cobb angle	27.66° (11.77)	brace	Persian Bad Sobernheim Stress Questionnaire-Deformity and the Persian Bad Sobernheim Stress Questionnaire-Brace	The stress level of patients using a brace was higher than in patients facing only the deformity (12.08 vs. 15.38, *p* < 0.01). The proportion of high stress level was higher in the case of stress caused by the brace in comparison with the stress caused by the deformity (27 vs. 7%, *p* < 0.01).
Kotwicki 2007 [21]	111	111	Adolescent idiopathic scoliosis evaluated by Cobb angle of the primary curve and Bunnell scoliometer angle of trunk rotation	42.8° (17.0°)	Physiotherapy, bracing and physiotherapy and preparation to surgical treatment	Bad Sobernheim Stress Questionnaire Deformity (BSSQ-Deformity) and the Bad Sobernheim Stress Questionnaire Brace	The braced patients revealed more stress when investigated for their braces than for their deformity (median = 18) comparing to BSSQ Brace (median = 9). The BSSQ deformity revealed a median of 17 points in patients managed with exercises, 18 in patients managed with a brace, and 12 in patients before spinal surgery correlation, between the total score of BSSQ deformity and the following parameters: Cobb angle (r = −0.34, *p* < 0.05).
Leszczewska 2012 [22]	73	73	Idiopathic scoliosis diagnosed on the basis of the X-ray	23.9 (17.7)	Physiotherapy and a combination of Chêneau Brace and physiotherapy	Bad Sobernheim Stress Questionnaire Deformity (BSSQ-Deformity) and the Bad Sobernheim Stress Questionnaire Brace	BSSQ-brace questionnaire’s mean score was 10, which indicates medium stress.
Zimoń 2018 [23]	63	63	Adolescent idiopathic scoliosis assessed by Cobb angle and angle vertebra rotation (AVR) based on A-P X-ray scans in the standing position in every studied patients	24.2 (10.1)	DoboMed approach with or without the use of Chêneau brace	Bad Sobernheim Stress Questionnaire Deformity (BSSQ-Deformity) and the Bad Sobernheim Stress Questionnaire Brace (BSSQ-Brace)	IS patients experienced low or moderate deformity-related stress (58.7% and 36.5%, respectively). In brace wearers, the orthosis-related stress was higher than the deformity-related stress (*p* < 0.0001). Patients with moderate to severe scoliosis (35–44°) exhibited significantly greater stress level (*p* = 0.038) compared to those with low to moderate scoliosis (16–24°).
Studies on the association between AIS and depression							
Lin 2019 [24]	208	112	A clinical diagnosis of adolescent idiopathic scoliosis and juvenile idiopathic scoliosis	31.4° (3.8) (before bracing) Juvenile Idiopathic Scoliosis angle: 29.8° (2.6°)	Brace	Zung Self-Rating Depression Scale and the Center for Epidemiological Studies Depression Scale for Children (CES-DC	Lower degree of depression in the JIS group (*p* < 0.0001) AIS Patient CES-DC significant mean Score: 38.3 ± 3.0 Females exhibited greater extent of depression (*p* < 0.05).
Chang 2016 [25]	8454	1409	Ninth revision of International Classification of Diseases Clinical Modification (ICD-9-CM) codes 737.X for scoliosis	NR	NR	through evaluation by psychiatry specialist on the basis of the criteria of Diagnostic and Statistical Manual of Mental Disorders (DSM-4)	The AHRs for age, gender, geographic region, osteoporosis, and spondylolisthesis of depression in patients with scoliosis was higher (AHR 1.95; 95% confidence interval (95% CI) 1.58–2.42; *p* < 0.001) than that of the controls during the 5-year follow-up. The middle age (41–65 years old) and young adults (18–40 years old) scoliosis patients had higher AHRs (middle-age: AHR 2.45; 95% CI 1.67–3.59; *p* < 0.05; young adult: AHR 1.99; 95% CI 1.41–2.82; *p* < 0.05); male subjects (HRs 2.45, 95% CI 1.59–3.78; *p* < 0.001) had a higher risk of depression than female subjects (HRs 1.83, 95% CI 1.43–2.34; *p* < 0.001).
Płaszewski 2014 [26]	144	68	Adolescent idiopathic scoliosis (74%) and early-onset idiopathic scoliosis(16%) screened at the Centre of Corrective and Compensatory Gymnastics	92%: 11–24° 8% 25–40° Mean cobb angle: 15.16° (6.44)	observation or exercise program	Beck Depression Inventory (BDI)	More scoliotic than nonscoliotic participants showing depressive symptoms (45% vs. 33% of subjects, respectively). The differences between the subjects with mild and moderate deformities were also significant (*p* < 0.05), with a greater tendency for depressive symptoms in subjects with milder deformities, more women than men exceeded 10 point threshold, which means severe depression.
Studies jointly examining the association of depression and anxiety with IS							
Wang 2019 [27]	149	64	Adolescent idiopathic scoliosis measured by two experienced surgeons based on the X-ray images, including the Cobb angle of the major curve, frontal balance, and shoulder height	mean major angle: 44.2 ± 18.9°	brace:29.7% no brace: 70.3%	Patient Health Questionnaire (PHQ-9) and a seven-item Generalized Anxiety Disorder scale (GAD-7)	Mean PHQ-9 and GAD-7 scores of the AIS patients were 4.0 ± 4.0 (0~16) and 2.9 ± 3.5 (0~19) Patients: Pmdd 7.9% (5/64) pGAD 3.2% (2/64)
Duramaz 2018 [28]	93	41	Adolescent idiopathic scoliosis radiographically measured according to the guidelines of the Scoliosis Research Society (SRS) Terminology Committee and Working Group	mean Cobb angle 52.5 (6.4) (preoperatively)	posterior instrumentation and fusion	Children’s Depression Inventory (CDI) state-trait Anxiety Inventory for Children (STAI-C)	Preoperative STAI-state and STAI-trait scores significantly higher in the study group compared with the control group (*p* = 0.001 and *p* = 0.001, respectively; *p* < 0.05). Postoperative STAI-state and STAI-trait scores decreased significantly in the study group compared with preoperative scores (*p* = 0.001 and *p* = 0.001, respectively; *p* < 0.05)
Wong 2019 [29]	987	987	Adolescent idiopathic scoliosis identified in medical and radiology records by a blinded investigator (PWHC)	Mild to moderate severity (Cobb angles of the largest curve) 44%: 21–30° 28%: 11–20° 18%: 31–40° 10%: >41°	observation, brace, posture training, surgery	Depression, Anxiety, and Stress Scale-21 (DASS-21)	2.0% of participants reported very severe depression and 3.2% reported anxiety. - AIS patients with back pain demonstrated clinically significant depression,(OR, 2.49; *p* = 0.03; 95% CI, 1.08–5.71)
Baird 2020 [30]	33	33	Establish diagnosis of adolescent idiopathic scoliosis	NR (severe scoliosis > 40° could be assumed by the type of treatment i.e., surgical correction)	surgery	Mood and Feelings Questionnaire: short version (SMFQ) social anxiety: three indicative questions, formulated from the expert opinion of the NICE panel	6/33 (18%, 95% CI: 7–35) had scores suggesting that further assessment for the potential diagnosis of depression was warranted. For the social anxiety questions, 19/32 of those surveyed (59%, 95% CI: 41–76) answered at least one question positively, indicating that further assessment for the diagnosis of social anxiety was indicated.
O Ryan 1999 [31]	28	28	Adolescent idiopathic scoliosis clinically assessed in an orthopaedic hospital	preoperatively: 53.71°	surgery	Hospital Anxiety and Depression Scale (HADS)	The cohort were experiencing an elevated level of anxiety but their mood was normal. Mean anxiety score was 7.4 (SD 3.84); 14 (60.9%) fell below the cut-off and 9 (39.1%) were above.
Sanders 2018 [32]	92	92	Adolescent idiopathic scoliosis clinically assessed	Observation group: 30.9 Surgical group: 56.6 Brace group: 33.2	observation, bracing, or surgery	Behavioral Assessment System for Children, Second Edition (BASC-2)	31.5% (29/92) of AIS patients indicated clinically significant psychological difficulty. The most concerning subscale from the self-report forms was anxiety, with 29% falling in the at-risk or clinically significant range.
Rullander 2016 [33]	37	37	Adolescent idiopathic scoliosis clinically assessed in four spine centres	Severe scoliosis (>45°) assumed by the type of treatment i.e., surgery	Surgery	Trauma Symptom Checklist for Children—Alternative version, Youth Self-Report and Kiddie Schedule for Affective Disorder and Schizophrenia	The higher preoperative scores showed significant *p*-values for anxiety/depression (*p* = 0.05) and internalizing (*p* = 0.05) on the YSR scale The level of pain correlated significantly with preoperative anger (*p* = 0.02), social problems (*p* = 0.01), and attention problems (*p* = 0.05).
Lee 2021 [34]	1,047,603 ± 34,534 (mean number in each dataset)	7409 ± 158 (mean number for each year)	Scoliosis defined by the ICD-10: M41.2 (other idiopathic scoliosis), M41.3 (thoracogenic scoliosis), M41.8 (other forms of scoliosis), and M41.9 (scoliosis, unspecified)	NR	NR	ICD-10 diagnostic codes: F3 (mood disorders), F4 (neurotic, stress-related, and somatoform disorders),) selected by a pediatrician (HWC) and an orthopedic surgeon (BHL)	7% of children with AIS had psychiatric disorders. In all 5 years, anxiety disorders accounted for the greatest prevalence. The adjusted ORs of psychiatric disorders in children with AIS ranged from 1.47 to 1.74 (95% CI, *p* < 0.001) in all 5 years.
Studies on the association between IS and eating disorders							
Alborghetti 2009 [35]	207	207	Adolescent idiopathic scoliosis evaluated indirectly through the kind of treatment and through the degree of vertebral column deviation	27.01 (only for a subset of the sample [i.e., *n* = 78])	physical exercises bracing or surgery) lasting for at least three years	eating disorders module of the SCIDI (First, Spizer, Gibbon, and Wiliams, 1997)—a semi structured clinicalinterview for DSM IV Axis I diagnoses—to assess EDs (full diagnosis criteria were used	EDs more likely in women suffering from scoliosis rather than in the Italian female population 9.2% (*n* = 19) for AN, 7.7% (*n* = 16) for BN and 5.3% (*n* = 11) for EDNOS (*p* < 0.001, *p* < 0.001, *p* < 0.05, respectively).
Smith 2002 [36]	44	44	NR	NR	NR	the body mass criteria of the International Classification of Diseases for eating disorders (ICD-10)	AIS patients were significantly lighter (*p* < 0.001) and had significantly lower BMI scores (*p* < 0.001); 25% of the series had BMI scores which were within the range considered to be an anorexic predictor in the multivariate regression model.
Zaina 2013 [37]	187 AIS patients 93 controls	187	Established diagnosis of idiopathic scoliosis: 24% juveniles, 76% adolescent type	26° (range 11–73°)	Braces (65%), specific exercises (12%) or observation (23%)	Italian validated questionnaire EAT-26	Only 3 (1.6%; 95% CI 0.2/3.4%) participants in the scoliosis group showed EAT-26 scores suggestive for eating disorders (20) versus 7 (7.5%; 95% CI 2.2/12.9%) in the school population; BMI was slightly but significantly lower for girls with AIS than controls (19 vs. 20 kg/m2, respectively)
Smith 2008 [38]	192	76	Idiopathic scoliosis	NR	NR	Eating Disorder Examination Questionnaire (EDE-Q) and weight and body mass index (weight [kg]/height [m(2)]) measurements	25% of the scoliosis group (19 were classified as clinically underweight, compared with 9% (7 participants) and 5% (2 participants) in the control and diabetes groups, respectively. The highest incidence of restrictive behavior was recorded in the scoliosis group, in 12 participants (15.8%). The scoliosis group was the only group to feature anorexia, with two cases (2.6%). No other scoliosis participants were classified with an eating disorder. The difference between the scoliosis and control groups was not significant (odds ratio 0.22, 95% confidence interval 0.02–1.22, *p* = 0.1).
Studies on the association between IS and personality pathology							
Leak 1992 [39]	80	40	Adolescent idiopathic scoliosis evaluated by cobb angle	26.35° (SD = 7.59)	brace	Interpersonal Reactivity Index (IRI) Millon Adolescent Personality Inventory (MAPI)	Scoliosis patients scored significantly higher than controls on passive-dependent functioning (M = 44.70 vs. M = 34.75). Higher personal distress scores for noncompliant (M = 14.82) than compliant (M = 11.48) patients (IRI) in the regime of bracing, with 9 of the 11 noncompliant patients (81.8%), but only 6 of the 29 compliant patients (20.7%), scoring in the pathological range in the sensitive scale of MAPI.
Misterska 2010 [40]	104	69	Clinical diagnosis of AIS following the criteria of the Scoliosis Research Society	27.7 (7.5)	35 treated conservatively; 34 treated surgically were treated with posterior correction and fusion (27 cases) or anterior correction and fusion (in 7 cases)	Erich Mittenecker and the Walter Toman Personality Test	Border-line score in the neurotic/non neurotic scale (M = 74.4) in the group treated surgically (VS brace treated: 63.3, healthy controls: 58.3) Braced group scored close to high intensity in the manic-non-manic scale (M = 73.0 vs. surgically treated group: 56.7 vs. health controls: 61.1). Positive correlation between time of brace application during a day, and the depressiveness and vegetative stability of the patients (*p* < 0.001).
Matsunaga 2005 [41]	145	145	Adolescent idiopathic scoliosis evaluated by Cobb angle	32°	brace	Maudsley Personality Inventory (MPI)	A total of 108 (74%) patients were rated as abnormal pattern on the Maudsley Personality Inventory tested 1 month after the start of therapy.
D’Agata 2017 [42]	43	43	Adolescent idiopathic scoliosis	32.9 (SD = 10.8)	braced and not treated (non-compliant to the brace)	16 Personality Factors-Adolescent Personality Questionnaire (16PF-APQ)	The lowest values were assessed for extroversion (M = 29.4, SD = 24.7) and self-reliance (M = 71, SD = 25.3).
Oh 2013 [43]	213	105	Adolescent idiopathic scoliosis confirmed by thoraco-lumbar standing radiographs (TLSR) during the conscription process	Mild severity (10–25°)and moderate to severe (25–40°, >40°)	NR	Korean military multiphasic personality inventory test (KMPI)	In the neurosis set, all the scales of anxiety, depression, and somatization were significantly increased in the AIS group (*p* = 0.010, 0.003 and 0.002, respectively). In the personality disorder scales showed a significantly increase in the AIS group (*p* = 0.001).
Studies on the association between AIS and psychosis							
Malmqvist 2019 [44]	373,902 (patients and controls)	3702	Adolescent idiopathic scoliosis identified in the NPR using the Swedish version of the 10th revision of International Classification of Disease (ICD-10-SE) with code M41.1/M41..2 for all cases of IS 1% Juvenile idiopathic scoliosis	NR	NR	10th revision of International Classification of Disease (ICD-10-SE) with code F20eF29 for both inpatients and outpatients with a diagnosis of schizophrenia	0.7% of patients with IS developed schizophrenia versus 0.5% of controls (*p* = 0.04). The risk of schizophrenia was significantly higher in patients with IS (HR, 1.52; 95% CI, 1.03–2.23)
Oh 2013 [43]	213	105	AIS confirmed by thoraco-lumbar standing radiographs (TLSR) during the conscription process	Mild severity (10–25°)and moderate to severe (25–40°, >40°)	NR	Korean military multiphasic personality inventory test (KMPI)	Schizophrenia showed a significantly increase in the AIS group (*p* = 0.010) score: 50.79 10.64 vs. 47.43 7.98 in the normal volunteer group

## Data Availability

Data are included in the manuscript.
